# Small Nucleolar (Sno)RNA: Therapy Lays in Translation

**DOI:** 10.3390/ncrna9030035

**Published:** 2023-06-08

**Authors:** Ofri Rabany, Daphna Nachmani

**Affiliations:** Department of Genetics, The Silberman Institute of Life Sciences, The Hebrew University of Jerusalem, Edmond J. Safra Campus, Jerusalem 9190401, Israel

**Keywords:** snoRNAs, 2′-O-methylation, ribosome, therapy

## Abstract

The ribosome is one of the largest complexes in the cell. Adding to its complexity are more than 200 RNA modification sites present on ribosomal RNAs (rRNAs) in a single human ribosome. These modifications occur in functionally important regions of the rRNA molecule, and they are vital for ribosome function and proper gene expression. Until recent technological advancements, the study of rRNA modifications and their profiles has been extremely laborious, leaving many questions unanswered. Small nucleolar RNAs (snoRNAs) are non-coding RNAs that facilitate and dictate the specificity of rRNA modification deposition, making them an attractive target for ribosome modulation. Here, we propose that through the mapping of rRNA modification profiles, we can identify cell-specific modifications with high therapeutic potential. We also describe the challenges of achieving the targeting specificity needed to implement snoRNAs as therapeutic targets in cancers.

## 1. Introduction

DNA and histone modifications (epigenomics) have an established role in regulating gene expression and disease onset [1,2,3]. However, the role of RNA modifications (the epitranscriptome) in these processes has only been recently delineated [4,5,6].

With 80 proteins and 4 RNA molecules, the ribosome is one of the largest complexes in the cell [7]. Adding to its complexity are more than 200 RNA modification sites present on ribosomal RNAs in a single human ribosome. rRNA modifications occur in evolutionary conserved and functionally important regions of the rRNA molecule. Moreover, rRNA modifications are essential for the processing and secondary structure of rRNA, and hence they are vital for ribosome function and proper gene expression [8,9,10]. The two most common post-transcriptional modifications include ribose 2′-OH hydroxyl methylation (2´OMe) and the conversion of uridine to pseudouridine (pseudouridylation, or Ψ). Studies have shown that changes to 2′OMe and Ψ are linked to the translational capacity of ribosomes [11,12], cellular properties, such as antibiotic sensitivity and immune function, as well as the maintenance of cancer cells [10,13,14,15,16,17,18,19]. We have previously demonstrated that aberrant 2′OMe is the etiological basis for bone marrow failure in dyskeratosis congenita patients [20]. Hence, aberrant rRNA modifications play a crucial role in human physiology and disease.

Together, 2′OMe and Ψ comprise more than 95% of modified rRNA nucleotides, with more than 110 2′OMe and ~100 Ψ modified residues in a single ribosome [19,21,22,23,24]. 2′OMe and Ψ are mostly introduced by two classes of small nucleolar RNAs, box C/D and box H/ACA, respectively. These two classes of snoRNAs are defined by their structure and sequence motifs, which are required for their association within snoRNPs and guidance of the complex to the nucleotide intended for modification. Box C/D snoRNA are associated with snoRNPs containing methyltransferase fibrillarin (FBL), and box H/ACA snoRNA with pseudouridine synthase dyskerin (DKC1). As 2′OMe and Ψ installation is based on extensive base-pairing between a snoRNA and an rRNA, many snoRNAs facilitate the modification of a single site [21,22]. This exclusive interaction between a snorRNA and a modification site enables precise targeting of specific rRNA modification sites through perturbations of the related snoRNA.

Even though many RNA modifications have been known since the 1950s, due to technological limitations, their investigation has thus far been extremely laborious and mostly confined to a single modified site at a time. This hindered our understanding of their function. Moreover, the lack of genetic tools for research in vertebrates and/or mammalian models has restricted our capacity to explore their physiological function and relevance to human disease. In the past decade, we have witnessed a boom in technological advancements, making it possible to detect RNA modifications transcriptome-wide and with single nucleotide resolution. This technological leap enables us to revisit the contribution of rRNA 2′OMe and Ψ in human disease to reassess the therapeutic potential of snoRNAs.

Indeed, in recent years, aberrant snoRNA expression has been described in various malignancies. The description of this body of work has been extensively reviewed by others [25,26,27]. However, it is important to note that increased ribogenesis and high rRNA modification levels are a feature of malignancies. A possible explanation for this is the malignant cell’s need for the fast accumulation of biomass in order to facilitate division and a high proliferative rate. This dependence on high modification levels might serve as a liability of the cancer cell as we can use it to specifically target those cells. Due to their pivotal role in the installment of rRNA modifications, ribogenesis, and gene expression, snoRNAs have great therapeutic potential.

## 2. The Challenges

### 2.1. Specificity

SnoRNAs are ubiquitous and highly abundant RNAs [21,28]. So, how can one target the malignant cell with little to no harm to healthy cells?

**Figure 1:**
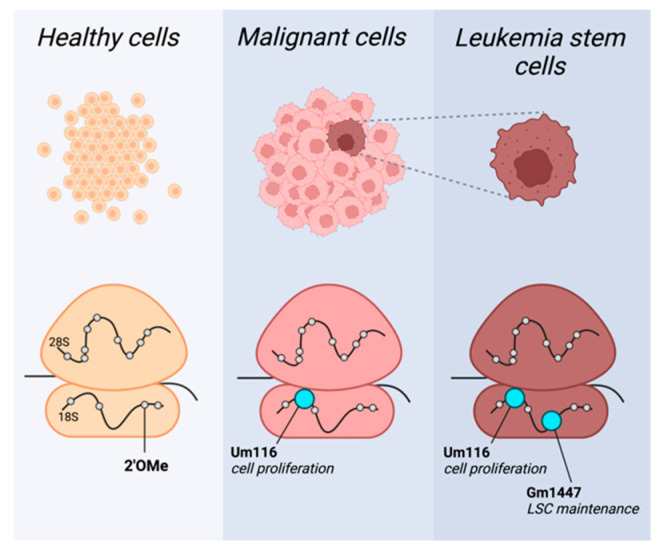
**2′OMe rRNA profiles provide specificity to target malignant cells.** Cell-type-specific rRNA 2′OMe profiles in healthy (yellow), malignant (pink), and leukemia stem cells (LSC; brown). Methylation pattern shifts in cancer, with specific sites highly methylated in leukemia blasts generally and in leukemia stem cells specifically, exemplified here by Um116 and Gm1447, respectively (depicted in cyan). This figure was created by BioRender.com.

Recent publications have demonstrated that some rRNA modifications occur in sub-stoichiometric levels [10,29]. This finding has overturned the notion that all rRNA modifications are constitutively present in ribosomes [23]. This also highlights nucleotide modifications as an important source of ribosomal heterogeneity, or cell-type-specific ribosomes. Together with the identification of high snoRNA levels in malignancies, this suggests that it is possible to identify cancer-specific rRNA modification patterns (Figure 1).

These cancer-specific modification profiles, once identified, will lead to the subsequent snoRNAs, which are crucial for the malignant cell. Those snoRNAs would have the highest potential as candidates for therapeutic targeting due to their ability to provide cancer specificity, and so their targeting will mostly affect cancer cells and not healthy, by standard, cells.

Recent works have already uncovered a few such snoRNA-dependent modification sites crucial for the maintenance of cancers. Zhou et al. revealed that rRNA 2′OMe modulates protein translation, affecting leukemia stem cell (LSC) phenotypes and propagation [30]. Their work shows a distinct methylation pattern of patient-derived acute myeloid leukemia (AML) cells compared to healthy hematopoietic cells. Furthermore, they identified a cluster of 2′OMe sites in which methylation correlated with the expression of LSC genes. LSCs are a small subset of self-renewing AML stem cells crucial for cancer’s propagation. Zhou et al. demonstrate that methylation level at these sites directs the leukemogenicity of AML cells, likely through the translation regulation of amino acid transporters and heightened amino acid metabolism needed for cell stemness. The authors then focus on a single modification site of the aforementioned cluster, 18S-Gm1447. By genetic manipulations of the snoRNA, which guides the site’s methylation, SNORD127, the authors demonstrate its role in the maintenance of LSC properties and leukemogenesis.

Additionally, Pauli and Liu et al. uncovered another single methylation site important for the proliferation of AML cell lines [15]. They performed a snoRNA screen to identify snoRNAs essential for leukemia cells’ proliferation. One of the top hits was SNORD42A, suggesting that this snoRNA is essential for the growth of leukemic cells. Importantly, SNORD42A was also found to be highly expressed in primary AML blasts. The knockout of SNORD42A reduces the methylation at 18S-Um116 while hindering the proliferation and colony formation ability of cells. It seems that a reduction in U116 methylation in SNORD42A knockout cells decreases the overall translation in the cells, as well as a decreased translation of specific proteins, most prominently ribosomal proteins.

Herein, we focus on leukemia; however, snoRNAs have been found to be implicated in additional cancers [31,32]. For example, it was found that in non-small cell lung carcinoma, the knockout of SNORA7A, SNORA7B, and SNORA65 and subsequent reduction in pseudouridylation resulted in the inhibition of the cancer cell’s proliferation, invasion, and migration [33]. Additionally, snoRNA U50, which mediates the methylation of C2848 in 28 S rRNA, appears to be downregulated in several cancers including breast, prostate, and colorectal. U50 inhibits cell colony formation and is suggested to be a tumor-suppressor-like gene [34,35,36].

The above-mentioned examples demonstrate the central role of even a single modification site on rRNAs in regulating malignancies. The need of the malignant cell to tailor its ribosome and achieve a high proliferative rate becomes a liability as it generates specificity. Thus, the identification of tumor-specific modification profiles may expose new therapeutic targets and pose as a promising avenue for cancer therapy.

### 2.2. Targeting SnoRNAs

Several techniques have been developed to target cellular RNAs. Here, we discuss two.

#### 2.2.1. Antisense Oligonucleotides (ASOs)

ASOs are single-stranded deoxy-oligonucleotides, usually ~20 nt in length, which are designed to bind specifically to the target RNA and modulate its abundance. ASOs act through several mechanisms of action. Regarding the regulation of snoRNA abundance, we bring forth two such mechanisms.

##### RNase H1-Mediated Degradation

Ribonuclease H1, or RNase H1, resides in nuclei [37,38] and plays a role in maintaining chromosomal stability by removing RNA-DNA hybrids [39]. ASOs target RNA, forming ASO (DNA)-RNA heteroduplexes, which act as substrates for RNase enzymes [40]. A specific design of ASOs, in which the central region is made of unmodified nucleotides, named Gapmers, is used to this end. In Gapmers, the incorporation of flanking modified nucleotides increases the ASO/Gapmer binding affinity, and the unmodified nucleotides promote RNase H1 activity. Many FDA-approved ASO drugs act through the activation of RNases [41,42,43]. Using this mode of action ASO treatment will lead to reduced snoRNA abundance and low rRNA modification of the specific site.

##### Modulation of Splice Sites

Most snoRNAs are encoded from the introns of host genes [21]. The host genes are transcribed by RNA polymerase II, and after splicing occurs, the excised intron undergoes additional processing steps to produce the mature snoRNA [44]. Thus, the modulation of splicing constitutes a natural tactic to regulate snoRNA abundance. ASOs can also exert their function by redirecting splicing, leading to exon-inclusion or exclusion [45,46,47,48]. If ASOs are directed against a snoRNA host gene, they will affect snoRNA abundance. An example of the benefits of this mode of action by ASOs would be the targeting of a mutated beta-globin pre-mRNA to produce splice variant mRNA that restored hemoglobin production [45,48].

#### 2.2.2. Small Molecules Interacting with RNA

**Figure 2:**
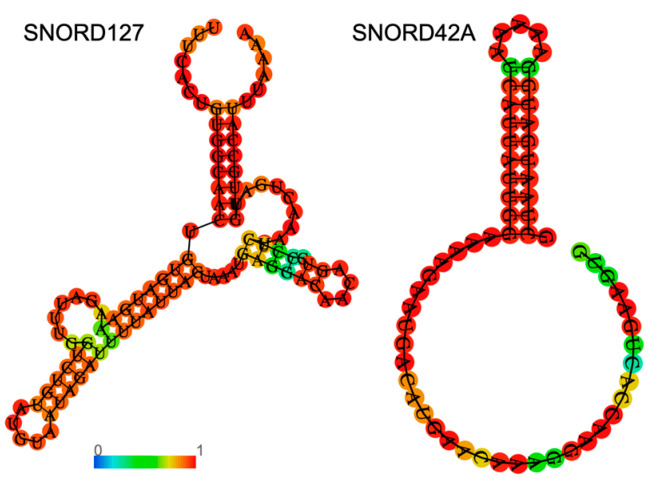
**Examples of specific secondary structures of C/D box snoRNAs**. Shown in the figure are predictions for the secondary structures of human SNORD127 (**left**) and SNORD42A (**right**), which were previously discussed. An RNA fold web server was used for structure prediction. The color of the nucleotides represents base-pair probability according to the color bar.

An interesting and promising alternative to sequence-specific targeting is structure-specific small molecules that bind to structures in the RNA molecule. Small molecules can be designed to lead to the degradation of their target, thus modulating their targets’ function. Small molecules are attractive therapeutic agents as they have low molecular weight and are less charged. These traits facilitate improved delivery efficiency compared with ASOs. Importantly, small molecules have been shown to be bioactive and tolerated in multiple models [49,50]. Importantly, while snoRNAs from the same family, either C/D or H/ACA box, share consensus sequences, snoRNAs can differ significantly in their secondary structure (Figure 2). These differences enable specific targeting of the desired snoRNA by custom-designed small molecules.

To identify potential small molecules in a high-throughput approach, RNA motif libraries are used to screen for small molecules that bind to a certain motif with high specificity. As these libraries are modular, they enable diverse experimental designs where one can either screen for the binding of many motifs or, alternatively, focus on specific motif families. Various mechanisms of action have been demonstrated by small molecules; for example, direct binding of the target RNA and its neutralization. Other small molecules have been designed to recruit endogenous nucleases, leading to the degradation of the target RNA [51,52]. The selectivity of small molecules can be even further increased by designing them to bind multiple sites on the RNA molecule [52,53]. To this end, dimeric compounds have been developed to simultaneously target two adjacent motifs within the RNA target with a defined distance between the motifs, increasing specificity. With respect to snoRNAs, this approach especially holds high potential, as the function of snoRNAs is dependent on their strong secondary structure. Thus, taking advantage of their molecular properties by targeting snoRNAs with small molecules serves as an interesting new therapeutic avenue.

## 3. Perspective

Thanks to technological developments and the rise of the non-coding RNA field, snoRNAs are now appreciated as facilitators of many molecular processes. These include ribogenesis, translation regulation by rRNA modifications or miRNAs, splicing, and more [31,54]. Because of their multi-functional nature, it is not surprising to find aberrant snoRNA expression in many malignancies, making them attractive therapeutic targets.

The growing appreciation of cell-type-specific rRNA modification profiles [10,15,29] enables the identification of snoRNAs that will facilitate high specificity for targeted therapy of the cancer cell’s ribosome. With the improved mapping of cancer-specific rRNA modification patterns, more cancer-specific snoRNAs can be identified, and therapeutic agents may be designed to promote the targeting of cancer cells.
